# The Prognostic Impact of Combined Tumor-Infiltrating Lymphocytes and Pretreatment Blood Lymphocyte Percentage in Locally Advanced Nasopharyngeal Carcinoma

**DOI:** 10.3389/fonc.2021.788497

**Published:** 2022-01-18

**Authors:** Zhuochen Cai, Jiali Jiang, Laiji Huang, Yawei Yuan, Ronghui Zheng, Jiangyu Zhang, Wenze Qiu

**Affiliations:** ^1^ State Key Laboratory of Oncology in South China, Collaborative Innovation Center for Cancer Medicine, Guangdong Key Laboratory of Nasopharyngeal Carcinoma Diagnosis and Therapy, Sun Yat-sen University Cancer Center, Guangzhou, China; ^2^ Department of Nasopharyngeal Carcinoma, Sun Yat-sen University Cancer Center, Guangzhou, China; ^3^ Health Ward, Affiliated Cancer Hospital and Institute of Guangzhou Medical University, Guangzhou, China; ^4^ Department of Radiation Oncology, Affiliated Cancer Hospital and Institute of Guangzhou Medical University, Guangzhou, China; ^5^ Department of Pathology, Affiliated Cancer Hospital and Institute of Guangzhou Medical University, Guangzhou, China

**Keywords:** locally advanced, lymphocyte percentage, nasopharyngeal carcinoma, prognostic impact, tumor-infiltrating lymphocytes

## Abstract

**Purpose:**

To explore the prognostic impact of combined tumor-infiltrating lymphocytes (TILs) and pretreatment peripheral lymphocyte percentage (LYM%) among patients with locally advanced nasopharyngeal carcinoma (LA-NPC).

**Patients and Methods:**

TILs and pretreatment LYM% were retrospectively assessed in 253 LA-NPC patients who underwent chemoradiation therapy between January 2012 and December 2017. According to TILs and LYM% status, the patients were divided into three groups: high-risk group (HRG) (TILs–LYM% score = 0), middle-risk group (MRG) (TILs–LYM% score = 1), and low-risk group (LRG) (TILs–LYM% score = 2). The relationship between TILs level and LYM%, and also the associations of TILs–LYM% status with clinicopathological factors and survival, were evaluated.

**Results:**

As a continuous variable, LYM% was significantly higher in TILs-high group. High TILs or high LYM% alone was significantly related to better 3-year disease-free survival (DFS), overall survival (OS), distant metastasis-free survival (DMFS) and locoregional relapse-free survival (LRRFS), respectively. Kaplan–Meier analysis and log-rank tests also revealed significant decreases in DFS, OS, DMFS, and LRRFS among LA-NPC patients with TILs–LYM% score of 0, 1, and 2 (all *P <*0.05). Further multivariate analyses showed that TILs–LYM% score was an independent factor affecting survival of the patients, and HRG (TILs–LYM% score = 0) had increased hazard ratios (HRs) for disease (HR = 6.89, *P <*0.001), death (HR = 8.08, *P* = 0.008), distant metastasis (HR = 7.66, *P* = 0.001), and local relapse (HR = 5.18, *P* = 0.013) compared with LRG (TILs–LYM% score = 2). In receiver operating characteristics (ROC) analyses, TILs–LYM% score had a higher area under the ROC curve (AUC) for the prediction of DFS than did TILs or LYM% alone.

**Conclusions:**

A positive correlation was found between TILs level and pretreatment blood lymphocyte percentage. Moreover, TILs–LYM% score can be considered as a novel independent prognostic indicator of survival outcome among patients with LA-NPC.

## Introduction

Nasopharyngeal carcinoma (NPC) is a unique head and neck cancer when taking into consideration its special geographic distribution, with high incidence in Southern China, Epstein–Barr virus (EBV) associated etiology, and histology (1). Most nasopharyngeal cancer can be cured if detected at an early stage. However, because the primary anatomical site of tumor growth is located in a cryptic site and is asymptomatic at early stages, NPC patients were usually diagnosed at an advanced stage, resulting in delayed treatment and causing poor long-term prognosis (2). At present, the Tumor-Node-Metastasis (TNM) system has been the most relevant clinicopathological variable for the prognostication of the locally advanced NPC (LA-NPC) patients undergoing oncologic treatment (3, 4). Nevertheless, the comprehensive TNM framework is merely based on the locoregional tumor expansions of the primary tumor, neglecting the substantial tumor- and host-related biological differences. In consequence, new prognostic determinants which can reflect the biological and immunological heterogeneity of NPC are required to predict the clinical course of patients more reliably and precisely.

Evidence is accumulating that immunological status, an essential biological marker, has an important role in carcinogenesis and development of cancer (5, 6). Lymphocytes, namely, tumor-infiltrating lymphocytes (TILs) and those in the peripheral blood, make up one of the most crucial effector mechanisms in the immunity to cancer. TILs, which mainly consist of T cells, B cells, and natural killer cells, are the predominant type of infiltrating immune cells (7). TILs are regarded as a manifestation of the host immune responses to cancer cells, and the potential of TILs as prognostic parameters has been reported in several tumors, namely, breast cancer, colon cancer and melanoma (8–10). NPC is known as lymphoepithelial carcinoma owing to the presence of an abundant infiltration of nonmalignant lymphocytes, and increased TILs have been associated with longer survival in NPC (11–14). On the other hand, a high peripheral lymphocyte percentage (LYM%) prior to initial treatment was also reported as an independent favorable prognostic factor in various types of tumors, such as cervical cancer, colorectal cancer, and NPC (15–17). However, few studies have reported the correlation between the level of TILs and pretreatment blood lymphocyte percentage in patients with malignancies. Additionally, there is no data on the prognostic value of the combination of TILs and peripheral lymphocyte status in LA-NPC.

The primary objective of the current study was therefore to investigate the correlation between the level of TILs and pretreatment blood lymphocyte percentage in patients with LA-NPC. Our secondary objective was to achieve a robust understanding of the prognostic impact of combined TILs and peripheral lymphocyte status among this population.

## Materials and Methods

### Study Population

This retrospective study included consecutive patients treated with curative radiotherapy for LA-NPC between January 2012 and December 2017. Inclusion criteria were: (1) pathologically diagnosed with undifferentiated NPC at the Affiliated Cancer Hospital and Institute of Guangzhou Medical University; (2) stage III–IVA disease according to the 8th edition of the American Joint Committee on Cancer (AJCC) staging system; (3) chemotherapy or radiotherapy naïve; and (4) complete pretreatment history of hematological variables. Exclusion criteria were: (1) with previous or concomitant other malignant diseases; (2) with distant metastasis; (3) with a history of immunodeficiency disease; (4) with active hepatitis B or C infection; (5) with active tuberculosis infection; and (6) absence of detailed information or clinical data. The Institutional Ethical Review Boards of Affiliated Cancer Hospital and Institute of Guangzhou Medical University approved this study. All participants provided written informed consent prior to treatment. The Reporting Recommendations for Tumor marker Prognostic Studies (REMARK) criteria were followed in reporting the results of this study (18).

### Pathologic Assessment

Full-face hematoxilin and eosin stained (H&E) slides of tumor tissue were retrieved for the quantification of percentage of TILs. As there is no current consensus on TIL scoring in nasopharyngeal cancer, this study was performed according to the TIL scoring recommendations of the International TILs Working Group 2014 on breast cancer (19). All pathologic slides independently assessed by two dedicated pathologists, who were blinded to clinical information, namely, treatment allocation and outcomes. The inconsistent cases were reviewed until a final consensus scoring was obtained. According to their predominant area of infiltration, TILs was categorized into intratumoral TILs (itTILs) and stromal TILs (sTILs). The quantity of itTILs was defined as the percentage of tumor epithelial nests that contain infiltrating lymphocytes, which have a direct contact with tumor cells. The sTILs score represented the percentage of stromal areas occupied by infiltrating lymphocytes that does not directly contact with carcinoma cells. Both itTILs and sTILs are continuous parameters. TILs were assessed by combining itTILs and sTILs. We used a cutoff value of 10% for itTILs and 70% for sTILs and defined subgroups with a cutoff value based on the percentage of itTILs and sTILs (high-TILs: itTILs >10% and/or sTILs >70% vs. low-TILs: itTILs ≤10% and sTILs ≤70%). The cutoff points were chosen because it had been shown to discriminate prognosis in an earlier large-scale cohort study of NPC using the same measurement system (13).

### Examination of Blood Lymphocyte Status and Cut-Off Value

The complete blood count (CBC) was determined using a Sysmex XE-5000 automated hematology analyzer (Sysmex, Kobe, Japan). We collected the absolute lymphocyte count (ALC) and LYM% retrospectively from routine laboratory measurements within 4 weeks before the start of treatment. In the measurement of ALC and LYM%, the coefficients of variance were both <5.0%. The determination of optimal cut-off point of the ALC or LYM%, also known as Youden index, to predict disease-free survival (DFS) was done by performing receiver operating characteristic (ROC) curve analysis.

### Treatment Strategies

All patients received intensity-modulated radiation therapy (IMRT) as the primary treatment modality. Target volumes and corresponding prescribed doses were determined according to the Radiation Therapy Oncology Group (RTOG) IMRT protocols (20). Based on the treatment guidelines for NPC at our institution, concurrent chemoradiotherapy ± neoadjuvant/adjuvant chemotherapy (CCRT ± NAC/AC) was recommended to patients with stage III–IVA NPC. In the present study, 25 (9.9%) of the patients received CCRT only, 228 (90.1%) received CCRT + NAC/AC.

### Statistical Analyses

The Chi-square test was used to calculate the association of tissue and blood lymphocyte status with clinicopathological variables. Independent sample *t*-test was applied to compare the ALC or LYM% as a continuous variable between low and high TILs groups. The primary endpoint was disease-free survival (DFS); the secondary endpoints were overall survival (OS), locoregional relapse-free survival (LRRFS), and distant metastasis-free survival (DMFS). OS was calculated as the time from treatment to death from any cause, or patients were censored at last follow-up; DFS, until locoregional relapse, distant metastasis, or death from any cause; LRRFS, until first locoregional relapse; DMFS, until distant metastasis.

In order to analyze the prognostic value of combined TILs and blood lymphocyte percentage, we designed a TILs–LYM% score as follows: the patients with high TILs and high LYM% were assigned a score of 2 [low-risk group (LRG)]; the patients with only high TILs or high LYM% were assigned a score of 1 [middle-risk group (MRG)]; the patients with low TILs and low LYM% were assigned a score of 0 [high-risk group (HRG)]. Kaplan–Meier survival curves were drawn separately for DFS, OS, DMFS, and LRRFS events by TILs, LYM%, and our proposed TILs–LYM% score. The log-rank test was used to evaluate the statistical significance of differences between the survival curves. Prognostic indicators were assessed using univariate and multivariate analyses (Cox proportional hazard regression model). Inter-rater and intra-rater agreements of itTILs and sTILs evaluation were calculated applying the Cohen Kappa coefficient (k). The prognosis value of TILs, LYM% and the combination were compared by area under the curve (AUC) values with Medcalc (Ostend, Belgium).

All statistical tests were two-sided and *P <*0.05 indicated statistical significance. Statistical analyses were performed using Statistical Package for the Social Sciences (SPSS) version 25.0 (IBM, Armonk, NY, USA).

## Results

### Demographical Features and Their Relationships With TILs and Blood Lymphocyte Status

**Figure 1:**
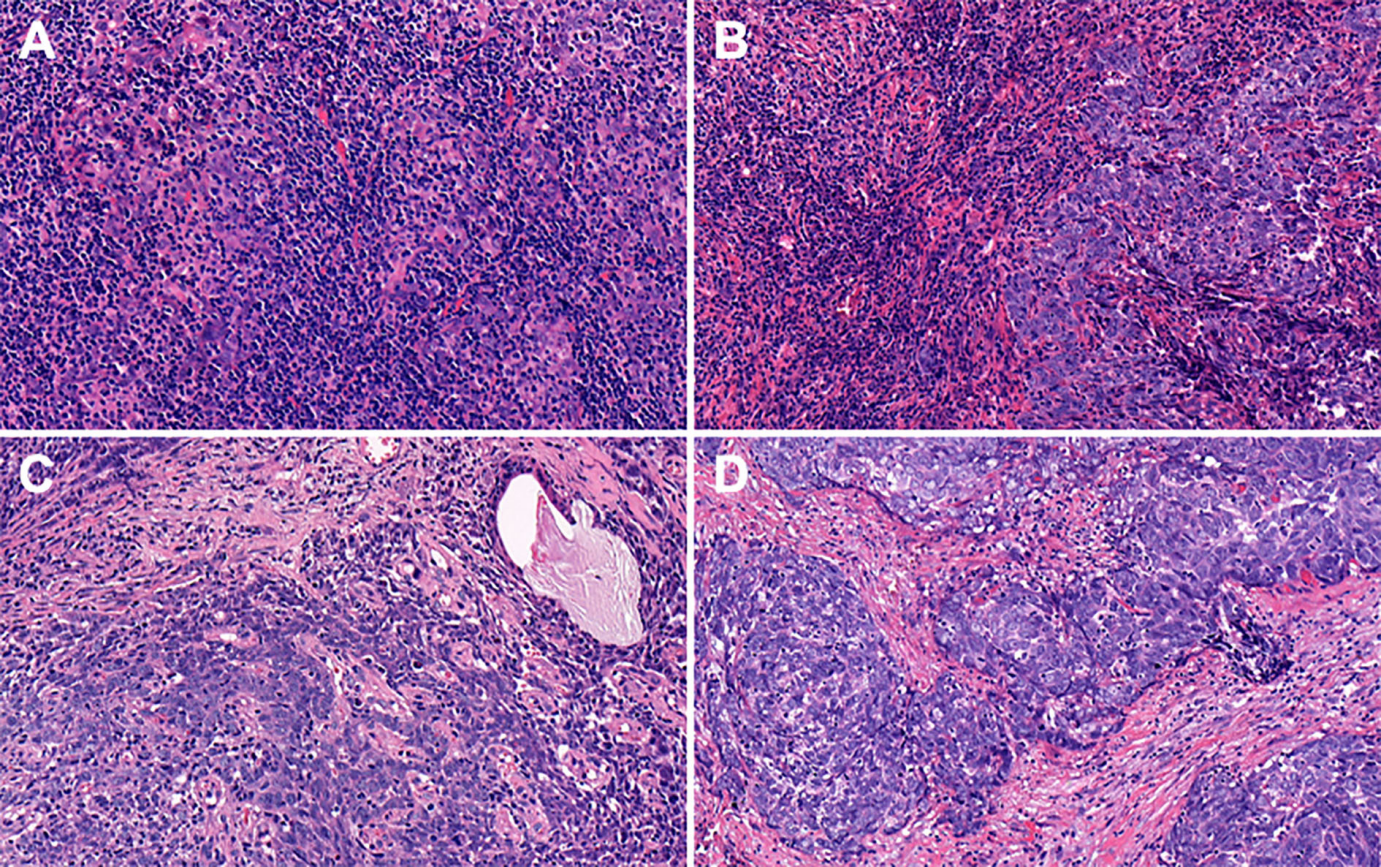
Representative features of hematoxylin and eosin (H&E) stained tumor sections with different degrees of itTILs and sTILs in nasopharyngeal carcinoma. **(A)** itTILs >10% and sTILs >70%. **(B)** itTILs ≤10% and sTILs >70%. **(C)** itTILs >10% and sTILs ≤70%. **(D)** itTILs ≤10% and sTILs ≤70%. itTILs, intratumoral tumor-infiltrating lymphocytes; sTILs, stromal tumor-infiltrating lymphocytes.

**Figure 2:**
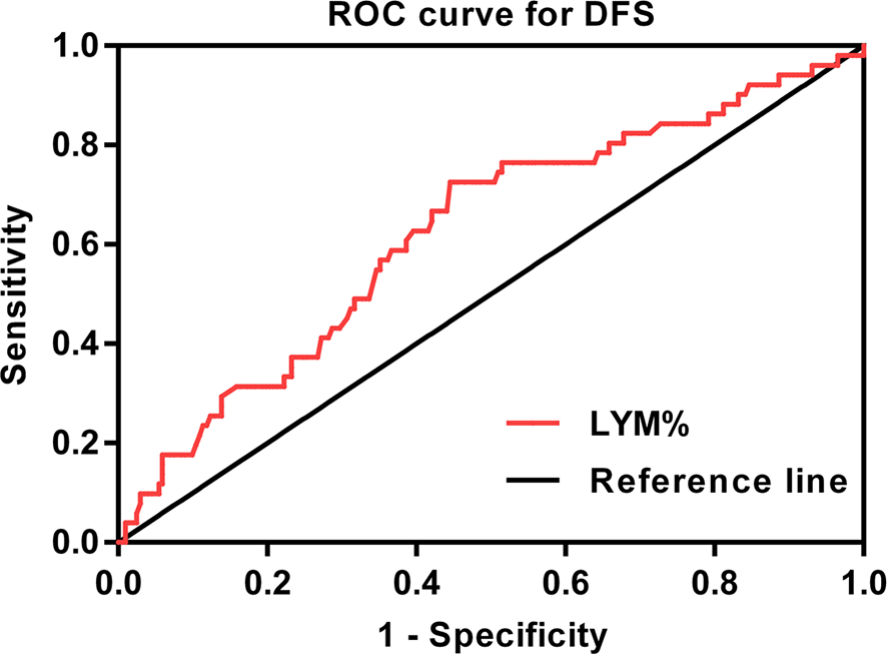
ROC curve for the pretreatment LYM% to predict DFS (AUC = 0.625). ROC, receiver operating characteristic; DFS, disease-free survival; LYM%, lymphocyte percentage; AUC, area under the curve.

**Figure 3:**
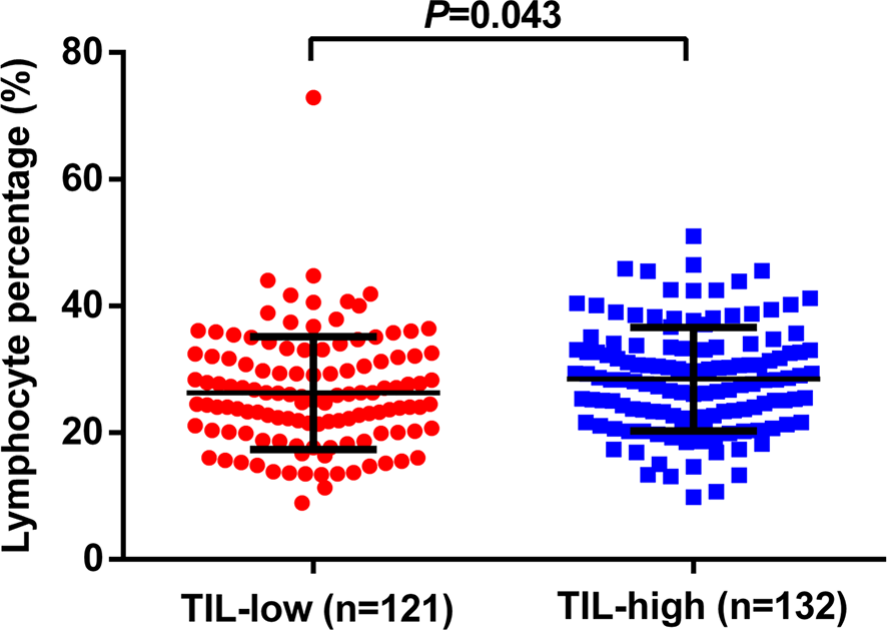
Relationship between TILs level and the pretreatment LYM%. TILs, tumor-infiltrating lymphocytes; LYM%, lymphocyte percentage.

By combining itTILs and sTILs, TILs were used to classify patients into different subpopulations, which allowed assessment of their function as a prognostic indicator. In our study cohort, 121 (47.8%) patients were defined as having low-TILs, and 132 (52.2%) patients as having high-TILs. The kappa coefficients for itTILs and sTILs were 0.80 and 0.75 for the inter-rater, and 0.85 and 0.81 for the intra-rater assessment respectively. **Figure 1** shows the histopathologic examples of lymphocytic infiltration. At the time of diagnosis, 80.2% (203/253) of patients with LA-NPC had peripheral lymphocyte percentage within the normal limits (20.0–50.0%), 48 patients (19.0%) lower than the normal limits (<20.0%), and 2 patients (0.8%) higher than the normal limits (>50.0%). The mean and median lymphocyte percentages for the entire cohort were 27.3 and 26.3%, respectively, and the values ranged from 8.9 to 72.9%. Based on the ROC curve analysis results, the optimal blood lymphocyte percentage cut-off point that significantly associated with DFS was 27.0% (AUC = 0.625; **Figure 2**). Accordingly, the 253 patients were subdivided into the LYM%-low (LYM% ≤27.0%; n = 131) and LYM%-high (LYM% >27.0%; n = 122) groups. As a continuous variable, LYM% was significantly higher in TILs-high group (28.2 ± 8.2% vs. 26.3 ± 8.9%, *P* = 0.043, **Figure 3**).

The optimal cutoff points of ALC for DFS was 1.68 × 10^9^/L, with the AUC of ROC curve of merely 0.534 (**Supplementary Figure 1**), and there was no significant difference in 3-year DFS between ALC-high and low group (84.5 vs. 73.7%, *P* = 0.157, **Supplementary Figure 2**). The ALC in TILs-low group was similar to that in TILs-high group (1.92 ± 0.06 × 10^9^/L vs. 1.90 ± 0.06 × 10^9^/L, *P* = 0.871, **Supplementary Figure 3**).

The correlations between clinicopathological characteristics and the TILs or LYM% are shown in **Table 1**. There was no significant association of TILs or LYM% with age, gender, BMI, tumor stage, and treatment modality.

**Table 1:** Clinicopathologic characteristics of 253 patients according to TILs and pretreatment peripheral LYM%.

Characteristics	TILs			LYM%		
	Low (n = 121, %)	High (n = 132, %)	*P*	Low (n = 131, %)	High (n = 122, %)	*P*
Age			0.418			0.184
≤48 years	58 (47.9)	70 (53.0)		61 (46.6)	67 (54.9)	
>48 years	63 (52.1)	62 (49.6)		70 (53.4)	55 (45.1)	
Gender			0.793			0.444
Male	89 (73.6)	99 (75.0)		100 (76.3)	88 (72.1)	
Female	32 (26.4)	33 (25.0)		31 (23.7)	34 (27.9)	
BMI (kg/m^2^)			0.253			0.795
<23	71 (58.7)	68 (51.5)		73 (55.7)	66 (54.1)	
≥23	50 (41.3)	64 (48.5)		58 (44.3)	56 (45.9)	
T stage			0.849			0.397
T1–T2	29 (24.0)	33 (25.0)		35 (26.7)	27 (22.1)	
T3–T4	92 (76.0)	99 (75.0)		96 (73.3)	95 (77.9)	
N stage			0.468			0.450
N0–N1	23 (19.0)	30 (22.7)		25 (19.1)	28 (23.0)	
N2–N3	98 (81.0)	102 (77.3)		106 (80.9)	93 (77.0)	
TNM stage			0.956			0.414
III	72 (59.5)	79 (59.8)		75 (57.3)	76 (62.3)	
IV	49 (40.5)	53 (40.2)		56 (42.7)	46 (37.7)	
Treatment			0.212			0.214
CCRT alone	9 (7.4)	16 (12.1)		10 (7.6)	15 (12.3)	
CCRT + NAC/AC	112 (92.6)	116 (87.9)		121 (92.4)	107 (87.7)	

AC, adjuvant chemotherapy; BMI, body mass index; CCRT, concurrent chemoradiotherapy; LYM%, lymphocyte percentage; NAC, neoadjuvant chemotherapy; TILs, tumor-infiltrating lymphocytes.

### Combination of TILs and Peripheral Lymphocyte Status

The TILs–LYM% score, a novel lymphocyte-based prognostic marker, was calculated based on the TILs and the pretreatment peripheral lymphocyte percentage. According to this TILs–LYM% scoring system, patients were divided into low-risk (LRG; N = 72), middle-risk (MGR; N = 110), and high-risk (HRG; N = 71) groups. The baseline characteristics of the different groups are presented in **Table 2**. Patients with a medium TILs–LYM% score (MRG) had more advanced clinical stages than patients with a low or high TILs–LYM% score (LRG or HRG) (*P* = 0.003 and 0.017, respectively). Accordingly, patients in the MRG were more likely to receive CCRT + NAC/AC than patients in the LRG or HRG (*P* = 0.003 and 0.161, respectively). There was no significant difference in the age, gender, BMI, T stage, and N stage between the LRG, MRG, and HRG (**Table 2**).

**Table 2:** Clinicopathologic characteristics of 253 patients according to TILs–LYM% score.

Characteristics	LRG (n = 72, %)	MRG (n = 110, %)	HRG (n = 71, %)	*P*		
				LRG vs. MRG	LRG vs. HRG	MRG vs. HRG
Age				0.359	0.156	0.517
≤48 years	41 (56.9)	55 (50.0)	32 (45.1)			
>48 years	31 (43.1)	55 (50.0)	39 (54.9)			
Gender				0.140	0.754	0.262
Male	50 (69.4)	87 (79.1)	51 (71.8)			
Female	22 (30.6)	23 (20.9)	20 (28.2)			
BMI (kg/m^2^)				0.667	0.344	0.144
<23	39 (54.2)	56 (50.9)	44 (62.0)			
≥23	33 (45.8)	54 (49.1)	27 (38.0)			
T stage				0.313	0.671	0.139
T1–T2	19 (26.4)	22 (20.0)	21 (29.6)			
T3–T4	53 (73.6)	88 (80.0)	50 (70.4)			
N stage				0.825	0.304	0.185
N0–N1	16 (22.2)	26 (23.6)	11 (15.5)			
N2–N3	56 (77.8)	84 (76.4)	60 (84.5)			
TNM stage				0.003	0.551	0.017
III	51 (70.8)	53 (48.2)	47 (66.2)			
IV	21 (29.2)	57 (51.8)	24 (33.8)			
Treatment				0.003	0.158	0.161
CCRT alone	13 (18.1)	5 (4.5)	7 (9.9)			
CCRT + NAC/AC	59 (81.9)	105 (95.5)	64 (90.1)			

AC, adjuvant chemotherapy; BMI, body mass index; CCRT, concurrent chemoradiotherapy; HRG, high-risk group; LRG, low-risk group; LYM%, lymphocyte percentage; MRG, middle-risk group; NAC, neoadjuvant chemotherapy; TILs, tumor-infiltrating lymphocytes.

### Survival in Terms of TILs and LYM% Status in Patients With LA-NPC

In our cohort, the median follow-up interval was 36 months (range, 3–136 months). During the follow-up, 202 patients (79.8%) did well without any evidence of disease progression, whereas 36 (14.2%), 25 (9.9%), and 28 (11.1%) patients experienced distant metastasis, local relapse, and death, respectively.

**Figure 4:**
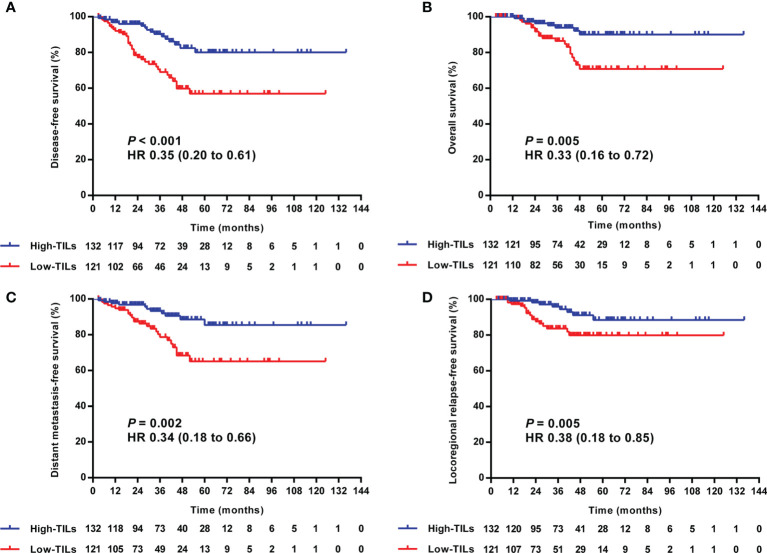
Kaplan–Meier curves of the DFS **(A)**, OS **(B)**, DMFS **(C)**, and LRRFS **(D)** of LA-NPC patients according to TILs level. DFS, disease-free survival; OS, overall survival; DMFS, distant metastasis-free survival; LRRFS, locoregional relapse-free survival; LA-NPC, locally advanced nasopharyngeal carcinoma; TILs, tumor-infiltrating lymphocytes.

In the univariate analysis, high-TILs were significantly related to better 3-year DFS, OS, DMFS, and LRRFS. The 3-year rates of DFS, OS, DMFS, and LRRFS for TILs-high vs. low group were 90.3 vs. 69.1% (*P <*0.001), 93.9 vs. 86.5% (*P* = 0.005), 93.3 vs. 78.7% (*P* = 0.002), and 95.9 vs. 83.6% (*P* = 0.018), respectively (**Table 3** and **Figure 4**).

**Table 3:** Univariate analysis of prognostic factors with locally advanced nasopharyngeal carcinoma.

Variate	3-year survival rate (%)							
	DFS	*P*	OS	*P*	DMFS	*P*	LRRFS	*P*
Age		0.499		0.067		0.889		0.920
≤48 years	79.4		94.0		85.1		89.8	
>48 years	81.7		86.4		88.4		90.5	
Gender		0.447		0.064		0.599		0.797
Male	78.9		89.3		85.2		90.2	
Female	84.7		93.7		90.8		89.7	
BMI (kg/m^2^)		0.959		0.300		0.939		0.545
<23	82.8		88.8		88.9		91.6	
≥23	76.9		92.5		83.0		88.2	
T stage		0.013		0.120		0.155		0.238
T1–T2	90.8		97.8		89.7		93.6	
T3–T4	77.1		88.0		85.5		89.0	
N stage		0.087		0.070		0.270		0.034
N0–N1	92.7		97.1		95.2		97.6	
N2–N3	77.1		88.7		84.2		88.1	
TNM stage		0.002		0.062		0.005		0.682
III	86.6		94.5		91.6		91.9	
IV	71.4		84.7		79.2		87.7	
Treatment		0.334		0.790		0.197		0.551
CCRT alone	83.1		86.5		91.6		91.5	
CCRT + NAC/AC	79.8		90.8		85.8		89.9	
TILs		<0.001		0.005		0.002		0.018
Low	69.1		86.5		78.7		83.6	
High	90.3		93.9		93.3		95.9	
LYM%		<0.001		0.005		0.003		0.049
Low	69.5		83.1		78.2		85.0	
High	92.0		98.2		95.3		95.5	
TILs–LYM% group		<0.001		0.001		<0.001		0.017
LRG	96.4		98.5		98.5		97.8	
MGR	83.6		92.2		88.2		92.6	
HRG	58.5		79.0		70.6		78.0	

AC, adjuvant chemotherapy; BMI, body mass index; CCRT, concurrent chemoradiotherapy; DFS, disease-free survival; DMFS, distant metastasis-free survival; HRG, high-risk group; LRG, low-risk group; LRRFS, locoregional relapse-free survival; LYM%, lymphocyte percentage; MGR, middle-risk group; NAC, neoadjuvant chemotherapy; OS, overall survival; TILs, tumor-infiltrating lymphocytes.

**Figure 5:**
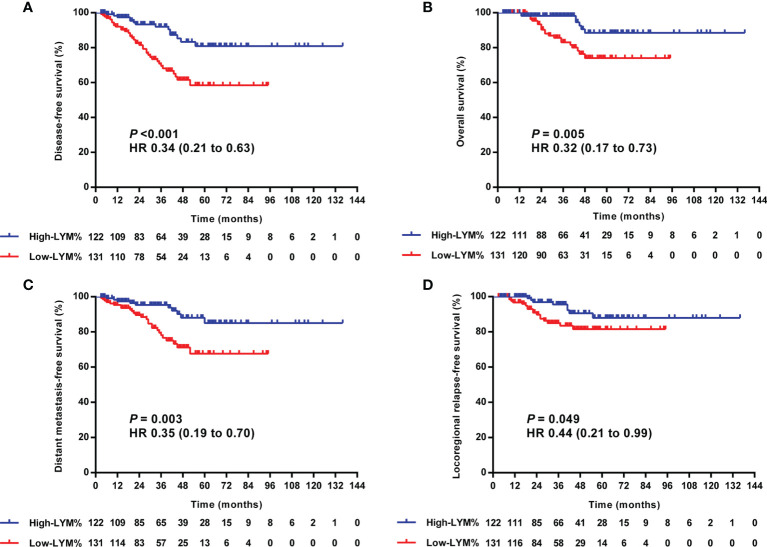
Kaplan–Meier curves of the DFS **(A)**, OS **(B)**, DMFS **(C)**, and LRRFS **(D)** of LA-NPC patients according to optimal cutoff point of LYM%. DFS, disease-free survival; OS, overall survival; DMFS, distant metastasis-free survival; LRRFS, locoregional relapse-free survival; LA-NPC, locally advanced nasopharyngeal carcinoma; LYM%, lymphocyte percentage.

Similarly, the patients in the LYM%-high group were more likely to experience superior survival than those in the LYM%-low group. The 3-year rates of DFS, OS, DMFS, and LRRFS for LYM%-high vs. low group were 92.0 vs. 69.5% (*P <*0.001), 98.2 vs. 83.1% (*P* = 0.005), 95.3 vs. 78.2% (*P* = 0.003), and 95.5 vs. 85.0% (*P* = 0.049), respectively (**Table 3** and **Figure 5**).

### Prognostic Values of the TILs–LYM% Score

**Figure 6:**
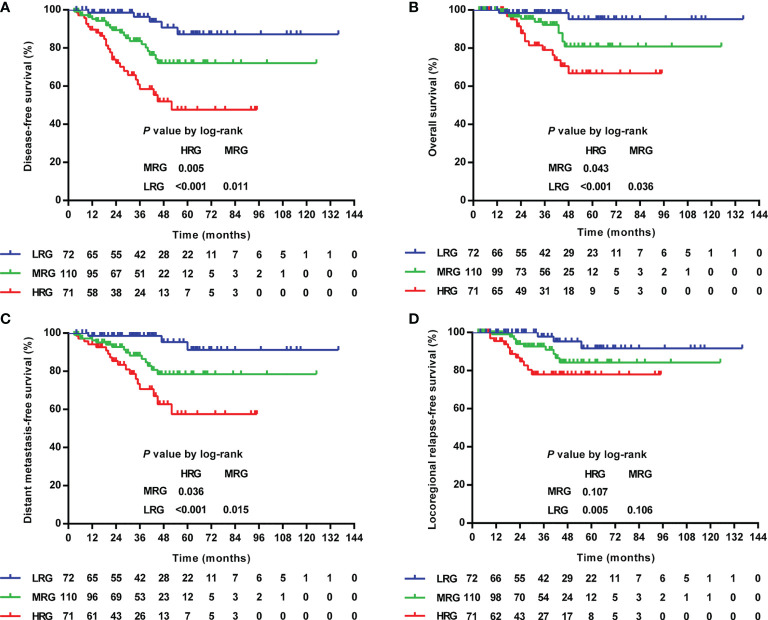
Kaplan–Meier curves of the DFS **(A)**, OS **(B)**, DMFS **(C)**, and LRRFS **(D)** of LA-NPC patients according to TILs–LYM% score. DFS, disease-free survival; OS, overall survival; DMFS, distant metastasis-free survival; LRRFS, locoregional relapse-free survival; LA-NPC, locally advanced nasopharyngeal carcinoma; TILs, tumor-infiltrating lymphocytes; LYM%, lymphocyte percentage; HRG, high-risk group (TILs–LYM% score = 0), MRG, middle-risk group (TILs–LYM% score = 1); LRG, low-risk group (TILs–LYM% score = 2).

Further exploration of the prognostic value in various TILs–LYM% scores showed that the higher the score, the lower the 3-year DFS (HRG/MGR/LRG: 96.4%/83.6%/58.5%, *P <*0.001), OS (HRG/MGR/LRG: 98.5%/92.2%/79.0%, *P* = 0.001), DMFS (HRG/MGR/LRG: 98.5%/88.2%/70.6%, *P <*0.001), and LRRFS (HRG/MGR/LRG: 97.8%/82.6%/78.0%, *P* = 0.017) (**Table 3** and **Figure 6**).

After adjustment for age, gender, BMI, tumor stage, and treatment modality, multivariate Cox analysis confirmed that HRG had increased hazard ratios (HRs) for disease (HR = 6.89, *P <*0.001), death (HR = 8.08, *P* = 0.008), distant metastasis (HR = 7.66, *P* = 0.001), and local relapse (HR = 5.18, *P* = 0.013), while MRG was not significantly prognostic for any survival outcomes (**Table 4**).

**Table 4:** Multivariate analysis of prognostic factors with locally advanced nasopharyngeal carcinoma.

		DFS			OS			DMFS			LRRFS	
Variate	HR	95%CI	*P*	HR	95%CI	*P*	HR	95%CI	*P*	HR	95%CI	*P*
Age (>48 vs. ≤48 year)	1.120	0.630–1.990	0.701	1.614	0.726–3.588	0.240	0.965	0.487–1.909	0.917	0.885	0.393–1.991	0.481
Gender (female vs. male)	0.801	0.402–1.594	0.527	0.383	0.114–1.291	0.121	0.839	0.373–1.890	0.672	0.824	0.321–2.117	0.839
Pretreatment BMI (≥23 vs. <23 kg/m^2^)	1.151	0.656–2.018	0.623	0.740	0.333–1.646	0.461	1.123	0.572–2.206	0.736	1.368	0.619–3.027	0.501
TNM stage (IV vs. III)	2.388	1.325–4.302	0.004	2.081	0.946–4.579	0.068	2.323	1.146–4.709	0.019	1.099	0.482–2.506	0.749
Treatment (CCRT + NAC/AC vs. CCRT only)	0.937	0.320–2.750	0.906	0.567	0.157–2.049	0.387	1.403	0.318–6.192	0.655	1.171	0.263–5.207	0.894
TILs–LYM% group			<0.001			0.008			0.001			0.040
LRG	1.000			1.000			1.000			1.000		
MGR	2.534	0.916–7.009	0.073	3.440	0.716–16.521	0.123	3.054	0.850–10.964	0.087	2.482	0.652–9.446	0.117
HRG	6.889	2.568–18.478	<0.001	8.075	1.736–37.561	0.008	7.663	2.189–26.830	0.001	5.179	1.420–18.884	0.013

AC, adjuvant chemotherapy; BMI, body mass index; CCRT, concurrent chemoradiotherapy; DFS, disease-free survival; DMFS, distant metastasis-free survival; HR, hazard ratio; HRG, high-risk group; LRG, low-risk group; LRRFS, locoregional relapse-free survival; LYM%, lymphocyte percentage; MGR, middle-risk group; NAC, neoadjuvant chemotherapy; OS, overall survival; TILs, tumor-infiltrating lymphocytes.

**Figure 7:**
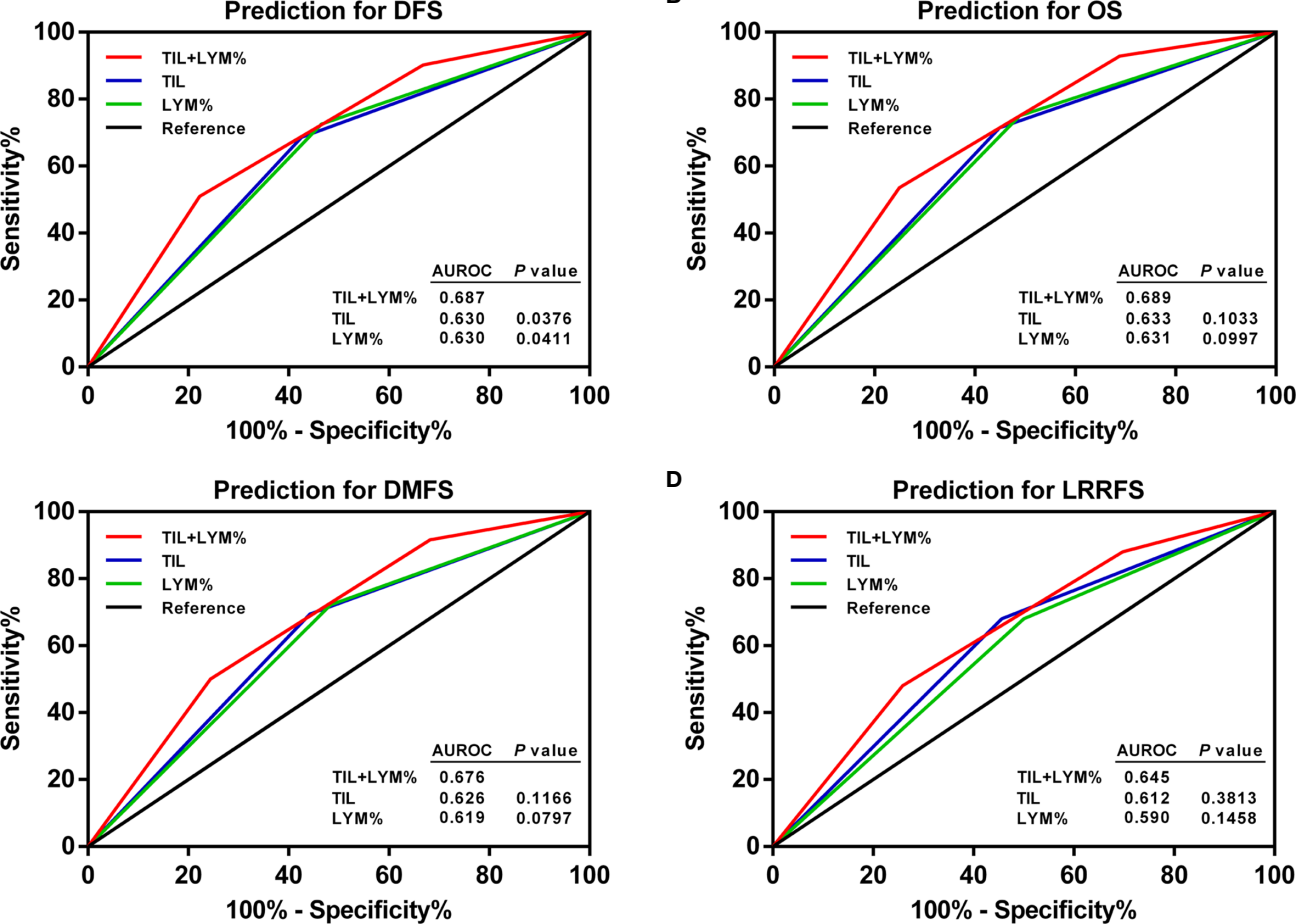
ROC curves of pretreatment TILs, LYM%, and TILs–LYM% score for the prediction of LA-NPC patients’ outcomes. **(A)** DFS, **(B)** OS, **(C)** DMFS, **(D)** LRRFS. DFS, disease-free survival; OS, overall survival; DMFS, distant metastasis-free survival; LRRFS, locoregional relapse-free survival; LA-NPC, locally advanced nasopharyngeal carcinoma; TILs, tumor-infiltrating lymphocytes; LYM%, lymphocyte percentage; ROC, receiver operating characteristic; AUROC, area under the receiver operating characteristic.

ROC curve analysis was applied to assess the effect of the TILs, LYM%, and TILs–LYM% score on the prognosis. Although the AUCs for TILs–LYM% scores with respect to DFS, OS, DMFS, and LRRFS were larger than those for TILs or LYM% alone, only the differences regarding DFS reached the statistical significance level (**Figure 7**).

## Discussion

Despite recent progress in the understanding of biological characteristics of NPC (21, 22), the routine prognostic risk assessment of NPC patients still depends on traditional clinicopathological prognostic variables, especially the TNM staging system (3, 4). However, this marker, mainly based on anatomical information, is difficult to reflect the immunological heterogeneity of the tumor. Here, we investigated the prognostic value of pretreatment TILs–LYM% score, a novel combined prognostic system, for 253 NPC patients undergoing chemoradiation therapy. To the best of our knowledge, this is the first study to reveal that pretreatment TILs–LYM% score is associated with survival outcome and can be considered as an independent indicator for better predicting disease-free survival of patients with LA-NPC.

The immune response to tumors, involving the interplay of several cell types of the innate and the adaptive immune systems, is complex and plays an important part in the progression of a variety of solid malignancies (23). In our study, high TILs are significantly associated with better survival outcome, which is in consistent with the results of the study of Wang (13). Peripheral blood lymphocytes are a non-specific yet commonly used bedside marker of cancer immunosurveillance (24). Our study suggests that pretreatment LYM% >27.0% is an independent positive prognostic factor on survival for LA-NPC patients.

Then we discovered that a positive correlation existed between the TILs and pretreatment LYM%. The mechanism underlying the relationship between TILs and LYM% is not well understood. Yet it is probably multifaceted and remains to be fully illustrated. As two components of the lymphocyte repertoire in patients with cancer, tumor-infiltrating lymphocytes and circulating lymphocytes are closely associated with each other. As shown recently, specific cytokines, chemokines, and adhesion molecules secreted by the cancer cells may be responsible for peripheral blood lymphocytes recruitment to tumor microenvironment, directly stimulate immune effector and stromal cells and enhance anti-tumor immunity (25). Positive correlations were found by Thomas et al. between both stromal and tumor nest CD8^+^ cells and circulating CD8^+^ levels (26). In our study, LYM% was significantly higher in high-TILs patients, which also supported the notion that infiltrating lymphocyte levels may to some extent reflect systemic lymphocyte levels.

In consideration of the findings mentioned above, a new scoring system by combining both TILs and LYM% (TILs–LYM% score) was developed, and risk groups were classified based on different scores. Patients in MRG had more advanced clinical stages and accordingly were more likely to receive CCRT + NAC/AC than patients in LRG. This finding is in agreement with the notion that growth of the primary tumor and metastatic spread were associated with decreased TILs densities or peripheral blood lymphocyte percentage in various human malignancies including NPC (13, 17, 27, 28). Patients in HRG, however, also had less stage IV disease than those in MRG. This paradoxical phenomenon may be explained by low number of evaluated patients; in addition, tumor invasion and host-immune reaction may not always evolve in a parallel way.

Based on the ROC curve analysis, the AUC value of the TILs–LYM% score for DFS surpassed those of TILs and LYM%, proving that TILs–LYM% score may achieve comparable prognostic performance on DFS that is even better than TILs or LYM% alone.

A lot of research has demonstrated that TILs are closely related to the crosstalk between tumor microenvironment components and immune system. Immune cells may be localized in the central zone of tumor, invasive margin, or tumor-adjacent stroma. The density, composition, functional state and organization of the leukocyte infiltrate of the tumor constitute the immune contexture, whereas cytokines and chemokines are involved in shaping it (29). Several studies suggest that solid tumors, namely, head and neck squamous cell carcinoma (HNSCC), showing an immune-desert phenotype (also called “cold tumors”) have a poor prognosis, for its lack of effective antitumor immune response, which is critical for limiting the tumor growth and reducing the risk of recurrences (30, 31). On the contrary, abundant lymphocyte infiltration would seem to have a great impact on preventing tumor recurrence in patients with NPC by altering the cancer-host immunity in the tumor microenvironment (12–14, 32).

Cancer cells have been detected in the peripheral blood in the patients with locally advanced or metastatic NPC, and the circulating tumor cells (CTCs) are considered as the origin for the metastasis and recurrence (33–35). However, the shedding of CTCs into peripheral blood is a necessary, but not sufficient condition for the formation of metastases (36). The interaction between tumor cells and peripheral immune cells plays a crucial role in tumor cell dissemination. Both higher neutrophil-dependent systemic inflammatory response and decreased lymphocyte mediated antitumor immune response will contribute to an elevated neutrophil–lymphocyte ratio (NLR), which is associated with shorter OS in metastatic castration-resistant prostate cancer patients with detectable CTCs (37). Thus, it is reasonable to speculate that a higher proportion of peripheral lymphocytes is prone to eliminating CTCs, leading to the reduction of metastatic relapse.

In theory, this lymphocyte-based prognostic marker is more persuading and shows stronger prognostic ability for predicting survival. However, we realized that TILs–LYM% score did not have significant advantages over TILs or LYM% alone on predicting DMFS, LRRFS and OS. While MRG is significantly associated with inferior DMFS, DFS, and OS in univariate analysis, it lost its prognostic importance in multivariate analysis. Lack of prognostication of MRG might be attributable to more stage IV patients in this group, thereby masking its prognostic value, and decreasing the prognostic performance of TILs–LYM% score.

The correlation between survival outcomes and tumor-infiltrating lymphocytes and circulating lymphocytes is warranted for further investigation in NPC patients, although both TILs and LYM% are easily available in the clinic. The TILs–LYM% score consists of TILs level and also defines the cutoff value of peripheral blood lymphocytes percentage before anti-cancer treatment, resulting in a particularly relevant multidimensional indicator with comprehensive prognostic information.

Immune checkpoint inhibitors (ICI), such as anti-PD-1 agents, have become part of the standard of care for treatment of recurrent or metastatic nasopharyngeal carcinoma (RM-NPC) (38, 39). Predictive biomarkers are needed to identify candidates who may benefit from anti-PD-1 treatments. TILs and peripheral blood lymphocyte were major players in the ICI mechanism of action, and they were found to be predictors for survival in patients with lung cancer on anti-PD-1 therapy (40, 41). Whether these two factors or their combination has prognostic impact on RM-NPC in the era of immunotherapy remains to be investigated.

A few limitations of our work ought to be highlighted. First, it was a retrospective cohort study with a small population from the endemic region, which might have led to bias and limited the generalizability of the findings. Second, the follow-up time was relatively short. Third, plasma Epstein–Barr virus (EBV) DNA information was not included because this blood test is not performed routinely in all our NPC patients.

## Conclusions

A positive correlation was found between TILs level and pretreatment blood lymphocyte percentage. Moreover, TILs–LYM% score can be considered as a novel independent prognostic indicator of survival outcome among patients with locally advanced NPC.

## Data Availability Statement

The raw data supporting the conclusions of this article will be made available by the authors, without undue reservation.

## Ethics Statement

The studies involving human participants were reviewed and approved by The Institutional Ethical Review Boards of Affiliated Cancer Hospital and Institute of Guangzhou Medical University. The patients/participants provided their written informed consent to participate in this study.

## Author Contributions

WQ and JZ conceived, designed and supervised the study. LH, YY, and RZ collected and analyzed the data. ZC and JJ wrote the manuscript. All authors contributed to the article and approved the submitted version.

## Funding

This work was supported by grants from the National Natural Science Foundation of China (No. 82002858), Guangzhou Key Medical Discipline Construction Project and Key Clinical Technology of Guangzhou (2019ZD17).

## Conflict of Interest

The authors declare that the research was conducted in the absence of any commercial or financial relationships that could be construed as a potential conflict of interest.

The reviewer YQ-X declared a shared affiliation, with no collaboration, with one of the authors, ZC, to the handling editor at the time of the review.

## Publisher’s Note

All claims expressed in this article are solely those of the authors and do not necessarily represent those of their affiliated organizations, or those of the publisher, the editors and the reviewers. Any product that may be evaluated in this article, or claim that may be made by its manufacturer, is not guaranteed or endorsed by the publisher.

## Supplementary Material

Supplementary Figure 1 ROC curve for the pretreatment ALC to predict DFS (AUC=0.534). ROC, receiver operating characteristic; DFS, disease-free survival; ALC, absolute lymphocyte count; AUC, area under the curve.

Supplementary Figure 2 Relationship between TILs level and the pretreatment ALC. TILs, tumor-infiltrating lymphocytes; ALC, absolute lymphocyte count.

Supplementary Figure 3 Kaplan-Meier curves of the DFS of LA-NPC patients according to optimal cutoff point of ALC. DFS, disease-free survival; LA-NPC, locally advanced nasopharyngeal carcinoma; ALC, absolute lymphocyte count.
