# Supplementary figures and images for: The Prognostic Impact of Combined Tumor-Infiltrating Lymphocytes and Pretreatment Blood Lymphocyte Percentage in Locally Advanced Nasopharyngeal Carcinoma
Source: Front Oncol. 2022 Jan 18;11:788497. doi: 10.3389/fonc.2021.788497 (PMC8804347; doi:10.3389/fonc.2021.788497)

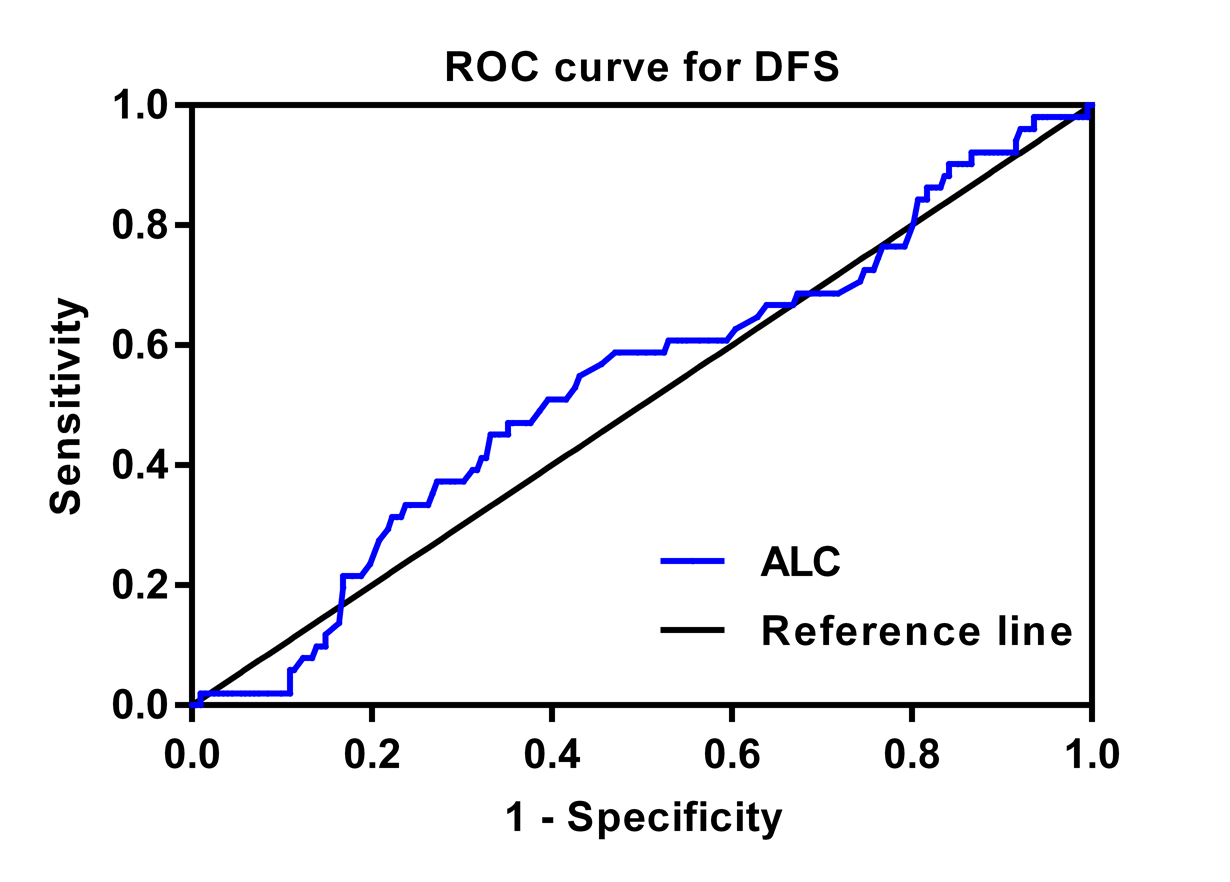

Supplement: Supplementary Figure 1 — ROC curve for the pretreatment ALC to predict DFS (AUC=0.534). ROC, receiver operating characteristic; DFS, disease-free survival; ALC, absolute lymphocyte count; AUC, area under the curve. [file Image_1.tif]

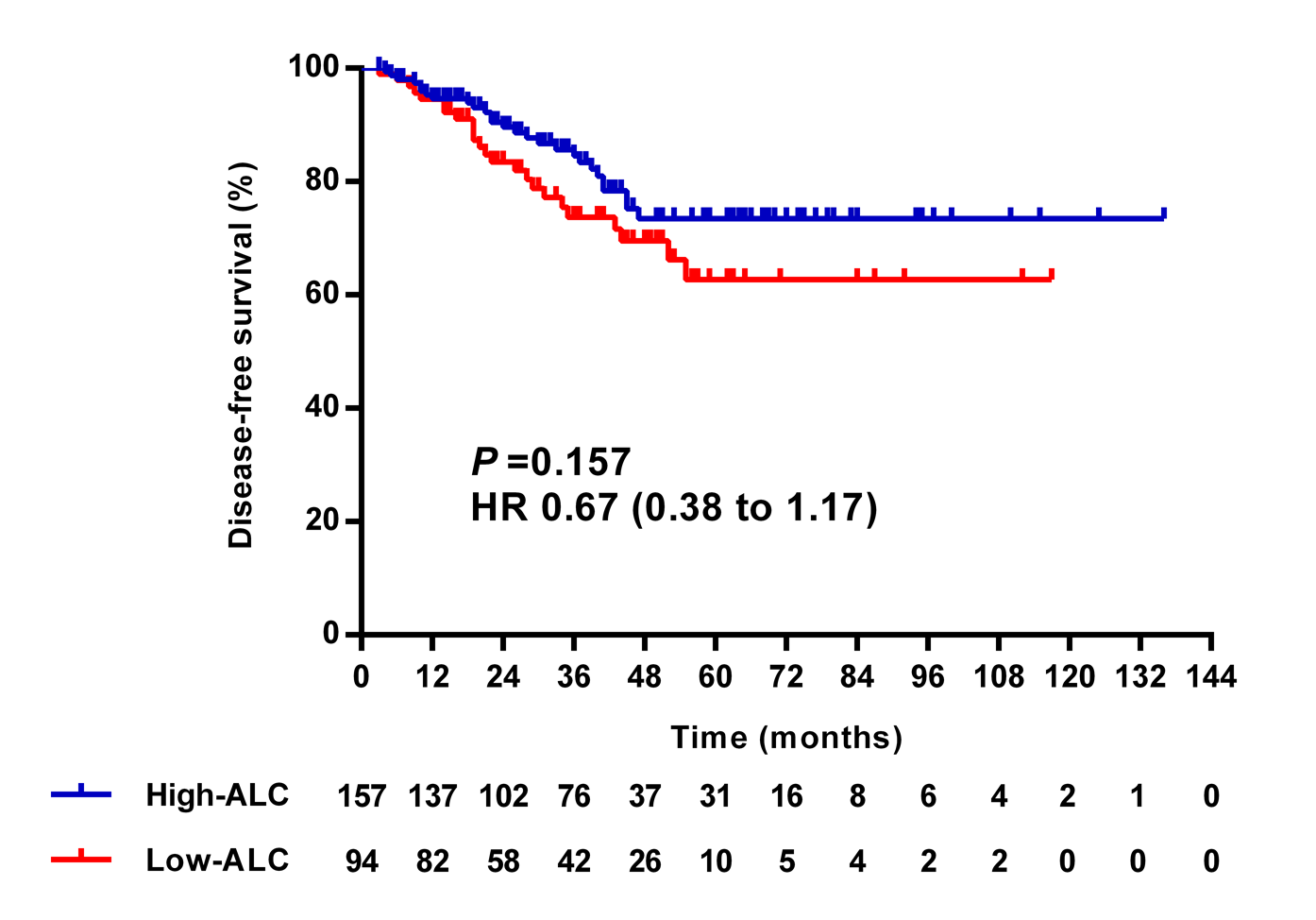

Supplement: Supplementary Figure 2 — Relationship between TILs level and the pretreatment ALC. TILs, tumor-infiltrating lymphocytes; ALC, absolute lymphocyte count. [file Image_2.tif]

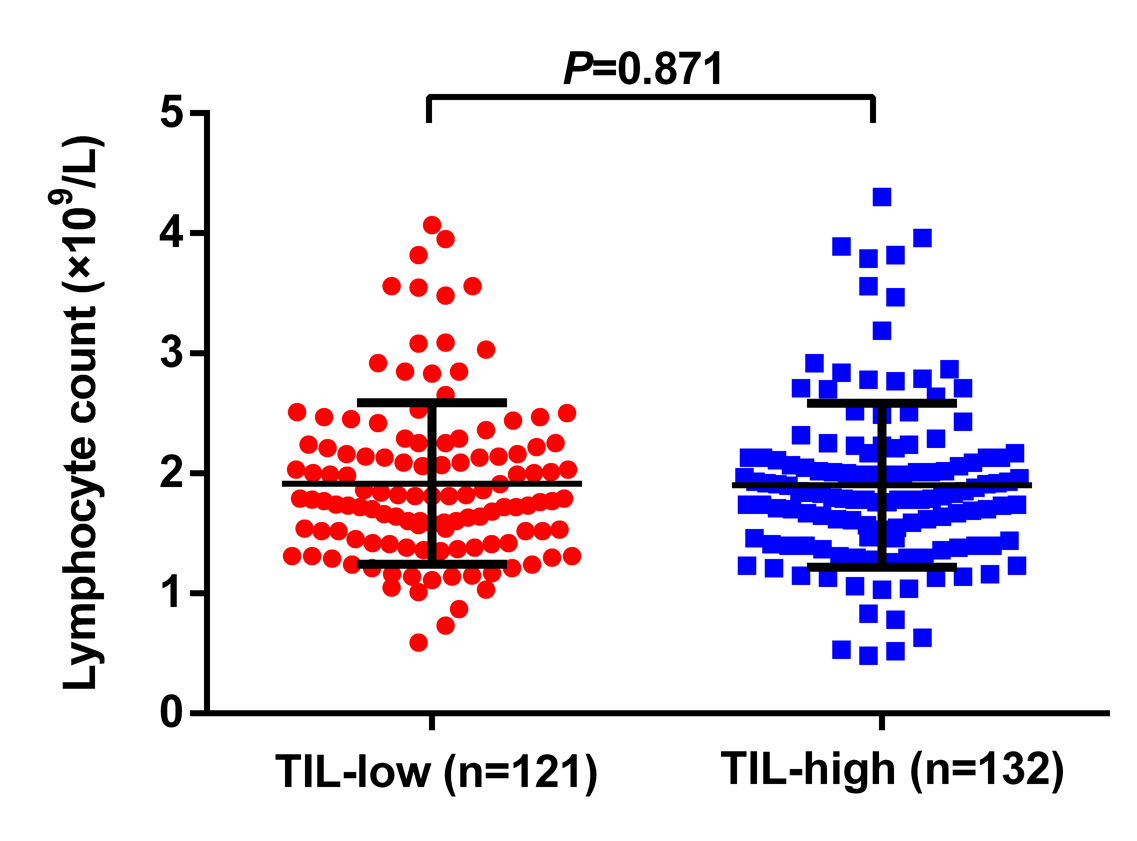

Supplement: Supplementary Figure 3 — Kaplan-Meier curves of the DFS of LA-NPC patients according to optimal cutoff point of ALC. DFS, disease-free survival; LA-NPC, locally advanced nasopharyngeal carcinoma; ALC, absolute lymphocyte count. [file Image_3.tif]
